# Complete mitochondrial genome of the common Pochard (*Aythya ferina*) from Ningxia Hui autonomous region, China

**DOI:** 10.1080/23802359.2021.2008820

**Published:** 2021-12-15

**Authors:** Hao Zhai, Dehuai Meng, Zongzhi Li, YuHui Si, Hongxian Yu, Liwei Teng, Zhensheng Liu

**Affiliations:** a College of Wildlife And Protected Area, Northeast Forestry University, Harbin, China; b Key Laboratory of Conservation Biology, National Forestry And Grassland Administration, Harbin, China

**Keywords:** Complete mitochondrial genome, *Aythya ferina*, Anatidae

## Abstract

We determined the whole mtDNA genome of the Common Pochard (*Aythya ferina*) in the Ningxia Hui Autonomous Region, China. The complete mitochondrial genome is 16,599 bp in length and consists of 13 protein-coding genes, 22 tRNA genes, 2 rRNA genes, and 1 control region (D-loop). The nucleotide composition is 29.34% A, 22.23% T, 15.66% G, and 32.77% C. Phylogenetic analysis results showed close genetic relationship between *A*. *ferina* and *Aythya americana*.

The Common Pochard (*Aythya ferina*), a widespread and common freshwater diving duck in the Palearctic region, was reclassified in 2015 from least concern to vulnerable IUCN status based on rapid declines throughout its range (Mischenko et al. 2020). The male has a red head and neck, red eyes, a long dark bill with a gray band, a black breast, and a gray back, and the female has a brown head and body and a narrower gray band on the bill (Madge and Burn 1988). Its breeding range extends from Western Europe through Central Asia to South-Central Siberia and Northern China (Kear 2005).

We sequenced the mitochondrial genome of *A*. *ferina*. Our samples were obtained from fresh muscle of *A*. *ferina* that died naturally in the Ningxia Hui Autonomous Region, China (105°57′E, 37°44′N) in October 2020. These specimens were stored in the College of Wildlife and Protected Area, Northeast Forestry University (No. HTQY2020). DNA sequencing was carried out using the MGISEQ-2000 platform (150-bp paired-end sequencing). The annotation and phylogenetic tree of the mitogenome sequence were determined using MITOS (Bernt et al. 2013) and MEGA 7, respectively.

The complete mitochondrial genome of *A*. *ferina* is 16,599 bp and consists of 37 genes, including 13 protein-coding genes (PCGs), 2 ribosomal RNA genes (12S ribosomal RNA and 16S ribosomal RNA), 22 tRNA genes, and 1 control region (D-loop). The nucleotide composition is 29.34% A, 22.23% T, 15.66% G, and 32.77% C. The total length of the 13 protein-coding genes is 10,555 bp; other than ND6, which is in the heavy strand (H strand), all of these genes are encoded on the same strand. Among the 13 protein-coding genes, the start codons are CCA in ND3, GTG in ND5, GTG in COX1 and COX2, and ATG in the remaining 9 protein-coding genes (ND1, ND2, ND4, ND4L, ND6, ATP6, ATP8, CYTB and COX3). The total length of the 22 tRNA genes is 1,543 bp, with genes ranging from 66 to 76 bp interspersed along the whole genome. The sequence lengths of the 12S RNA gene, the 16S RNA gene, and the D-loop region (control region) are 986, 1,604, and 1,019 bp, respectively. All of this information and the assembled sequences were submitted to GenBank under accession number MW337298. In addition, the raw sequencing data were deposited in SRA (SRA no. PRJNA699041).

The phylogenetic relationship was inferred by using the maximum likelihood method based on the Tamura-Nei model (Tamura and Nei 1993) and determined using MEGA7 (Kumar et al. 2016). The bootstrap consensus tree inferred from 1,000 replicates was taken to represent the analyzed taxa (Felsenstein 1985). The phylogenetic tree showed that *A. ferina* has a close phylogenetic relationship with *A. americana* (Figure 1).

**Figure 1.**
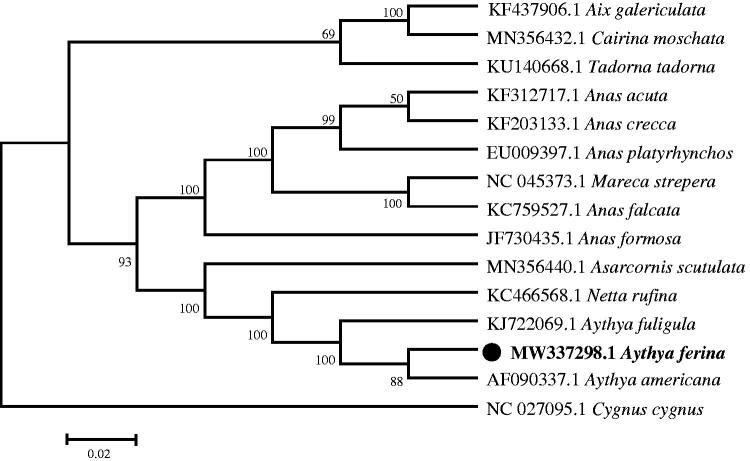
Phylogenetic tree generated using the Maximum Likelihood method based on complete mitochondrial genomes of 15 species.

## Data Availability

The data that support the findings of this study are openly available in GenBank of NCBI at https://www.ncbi.nlm.nih.gov/, reference number MW337298. The associated SRA number is PRJNA699041.
